# Metabolic alterations due to *IDH1* mutation in glioma: opening for therapeutic opportunities?

**DOI:** 10.1186/2051-5960-2-6

**Published:** 2014-01-09

**Authors:** Dana A N Mustafa, Sigrid M Swagemakers, Laura Buise, Peter J van der Spek, Johan M Kros

**Affiliations:** 1 Department of Pathology, Erasmus Medical Center, Dr. Molewaterplein 50, 3000 CA, Rotterdam, The Netherlands; 2 Departments of Bio-Informatics, Erasmus Medical Center, Dr. Molewaterplein 50, 3015 GE, Rotterdam, The Netherlands

## 

Recently heterozygous mutations in the active site of the enzyme isocitrate dehydrogenase 1 (IDH1) were discovered in glioblastomas [1]. In cohorts of glioma patients the *IDH1* mutation appeared to be a strong predictor of clinical outcome, overruling histological malignancy grade [2]. IDH1 is an enzyme of the tricarboxylic acid (TCA) cycle and is located in the cytosol, where it produces NADPH by transforming isocitrate into α-ketoglutarate. Because the mutant enzyme displays neomorphic activity through NADPH-dependent transformation of α-ketoglutarate into 2-hydroxyglutarate (2HG), the tumorigenic role of the increased levels of 2HG has become a target of speculation [3]. *IDH1* mutation alters the cellular metabolism and epigenetic phenotype influencing cellular proliferation. *IDH1* mutation infers increased levels of *D2HGDH* leading to the inhibition of DNA and histone demethylating enzymes, resulting in the glioma-CpG island phenotype [4]. Altered concentrations of pyruvate kinase M2 play also a role in histone modifications which are associated with the transcription of the proliferation-related cyclin D1 and c-MYC [5]. In addition, *IDH1* mutant cells show alterations in glutamine, fatty acid and citrate synthesis pathways, which all may have their influence on cellular proliferation [5].

The changes in *IDH1* function affect the glucose metabolism, which may explain the different biological behaviour of tumors with and without the *IDH1* mutation [6]. In order to detect these changes, we compared the expression levels of the genes participating in the TCA cycle and in the anaerobic glycolysis in 33 *IDH1* mutated samples (3 astrocytomas WHO grade II; 6 astrocytomas WHO grade III; 4 glioblastomas; 9 oligodendrogliomas WHO grade II; 11 oligodendrogliomas WHO grade III) and in 39 *IDH1* wild-type glioma samples (10 astrocytomas WHO grade III; 26 glioblastomas; 1 oligodendroglioma WHO grade II; 2 oligodendroglioma WHO grade III) and in four samples of normal brain (Table 1). We found expressional differences of 16/24 genes (Figure 1). The *IDH1* mutated cells seem to compensate for the low production of α-ketoglutarate by overexpressing *D2HGDH* and *L2HGDH* in the cytoplasm. They also overexpress *GLUD1*, which converts glutamate to α-ketoglutarate inside the mitochondria. In addition, we found that *IDH1* mutated cells overexpress *HIF1AN. The HIF1AN* gene inhibits *HIF1α.* Since *HIF1α* acts as an oxygen sensor that promotes angiogenesis, the formation of dysfunctional tumor vasculature is counteracted in *IDH1* mutated cells. Furthermore, the *IDH1* mutated cells overexpress the *LDHB* gene while cells without *IDH1* mutation overexpress the *LDHA* gene. The present results illustrate that tumor cells without *IDH1* mutation switch their energy production from a low rate of glycolysis followed by the TCA cycle to a high rate of glycolysis followed by aerobic glycolysis (*LDHA* up-regulation; Figure 1). The resulting lactate acid production causes tissue acidosis known as the Warburg effect. In invasive cancers, the pH of the extracellular space increases the infiltrative potential of the tumor cells [7]. In addition, normalization of the extracellular pH by alterations of the enzymatic actions of *LDHA* and *LDHB* influences the progression of cancer cells [8,9]. It may well be that glial tumor cells with *IDH1* mutation tend to correct their energy production through the TCA cycle by overexpressing *LDHB* (Figure 1). By doing so, they normalize the tissue pH which offers yet another explanation for the less aggressive biological behavior of the *IDH1* mutated gliomas.

**Table 1:** Percentages of glioma types and grades

**WHO grades**	**IDH1 mutation**	**IDH1 wild type**
A II	4%	0%
A III	8%	14%
GBM	6%	36%
O II	13%	1%
O III	15%	3%

Legends: *A II* low-grade astrocytoma (WHO grade 2), *A III* anaplastic astrocytoma (WHO grade 3), *GBM* glioblastoma (WHO grade 4), *O II* low-grade oligodendroglioma (WHO grade 2), *O III* anaplastic oligodendroglioma (WHO grade 3).

**Figure 1:**
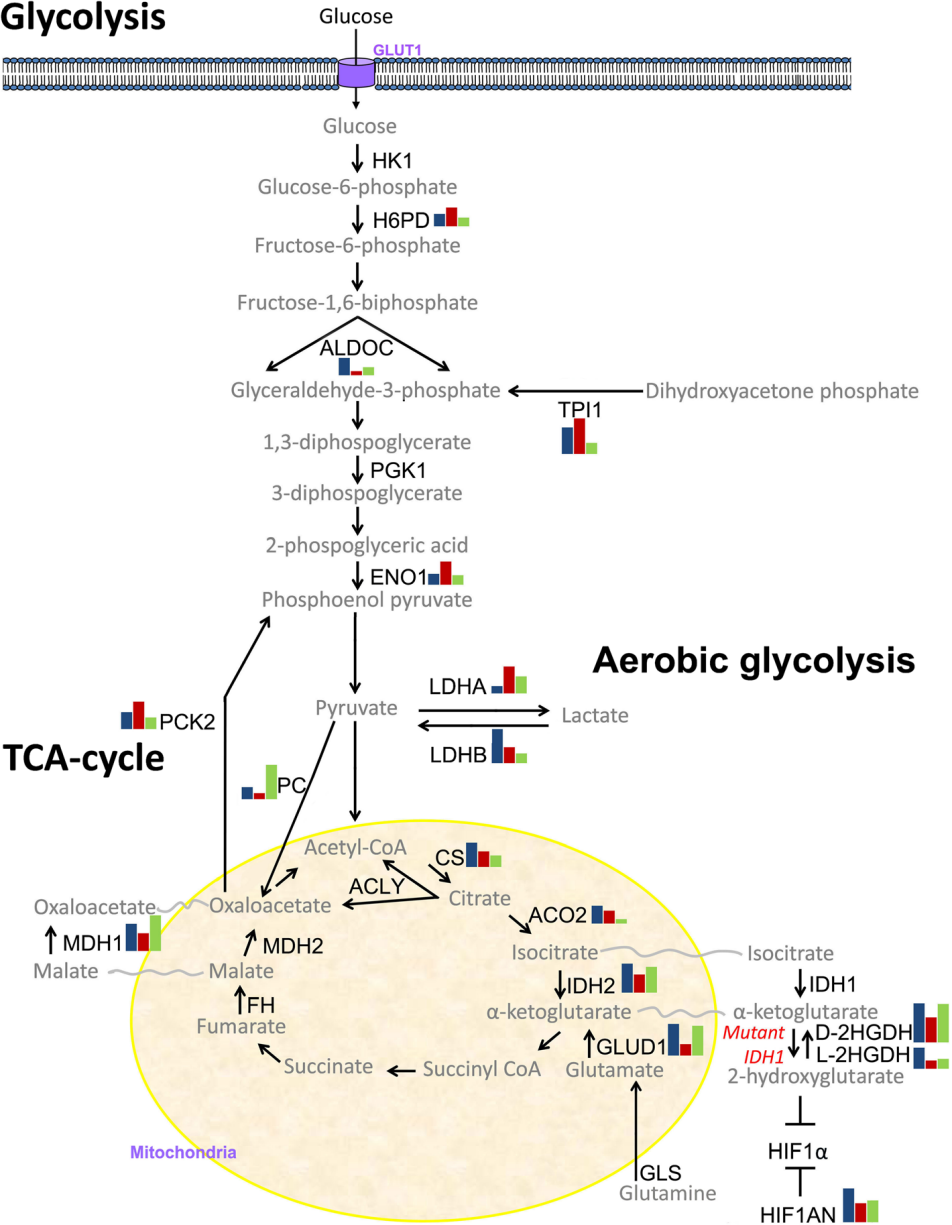
**Overview of alterations in glucose metabolism in the *IDH1 *mutated and *IDH1 *wild-type gliomas relative to glucose metabolism in normal brain.** The enzymes included in the analysis are shown in black. The bar diagrams show the averaged expressional levels of genes that were significantly differently expressed in the *IDH1* mutated gliomas (n = 33, blue bars on the left); *IDH1* wild-type (n = 39, red bars in the middle); and normal brain (n = 4, green bars on the right). Glycolysis leads to the production of pyruvate. In normal brain, pyruvate is converted to acetyl-CoA, which enters the TCA cycle in the mitochondria. But in the IDH1 wild-type tumors, pyruvate is metabolized to lactate (“the Warburg effect” in which the aerobic glycolysis serves as a quick energy source that results in tissue acidosis). To ensure the origin and quality of the tissues, all tissues were assessed by a qualified pathologist before isolation. IDH1 status was checked using IHC and the standard PCR test. Total RNA was isolated with the RNA-Bee (Campro, Veenendaal, The Netherlands). cDNA was prepared using the RevertAid H Minus First Strand cDNA synthesis kit (Fermentas, St Leon-Rot, Germany). The resulting cDNA preparations were analysed by real-time PCR with SYPR green master mix solution (Applied Biosystems, Nieuwerkerk a/d IJssel, The Netherlands). PCRs were performed in a 25 μL reaction volume in an Applied BioSystems 7900HT Fast Real-Time PCR system. Negative controls included minus RT and H_2_O-only samples, which were negative in all cases. Expression of *B2M*, *HPRT1* and *PBGD* was used as a reference to control sample loading and RNA quality. Differences in mRNA concentrations were determined using the *t-*test, with P < 0.01 being considered statistically significant. All statistical tests were two-sided.

We conclude that gliomas with *IDH1* mutation normalize their glucose metabolism, which appears to result in a slower tumor progression. Depending on the *IDH1* status of the tumor, specific interference with the glucose metabolism and aerobic glycolysis should therefore be considered for future therapeutic strategies.

## Competing interests

All authors declare that they have no competing interests.

## Authors’ contributions

DM and LB carried out the molecular genetic analyses; SS and PvdS carried out the data analysis and DM and JMK conceptualized and designed the study and wrote the manuscript. All authors read and approved the final manuscript.
